# Identification of Long-Term Care Facility Residence From Admission Notes Using Large Language Models

**DOI:** 10.1001/jamanetworkopen.2025.12032

**Published:** 2025-05-22

**Authors:** Katherine E. Goodman, Matthew L. Robinson, Seyed M. Shams, Pilar Beccar-Varela, Suiyini Fiawoo, Nathan Kwon, Jae Hyoung Lee, Abigail H. Vorsteg, Monica Taneja, Laurence S. Magder, Mark Sutherland, Scott Sorongon, Pranita D. Tamma, Daniel J. Morgan, Philip Resnik, Anthony D. Harris, Eili Y. Klein

**Affiliations:** 1 The University of Maryland School of Medicine, Baltimore; 2 The University of Maryland Institute for Health Computing, North Bethesda; 3 Division of Infectious Diseases, The Johns Hopkins School of Medicine, Baltimore, Maryland; 4 University of Maryland Medical System, Baltimore; 5 The Johns Hopkins School of Medicine, Baltimore, Maryland; 6 The University of Maryland, College Park

## Abstract

**Importance:**

An estimated half of all long-term care facility (LTCF) residents are colonized with antimicrobial-resistant organisms, and early identification of these patients on admission to acute care hospitals is a core strategy for preventing intrahospital spread. However, because LTCF exposure is not reliably captured in structured electronic health record data, LTCF-exposed patients routinely go undetected. Large language models (LLMs) offer a promising, but untested, opportunity for extracting this information from patient admission histories.

**Objective:**

To evaluate the performance of an LLM against human review for identifying recent LTCF exposure from identifiable patient admission histories.

**Design, Setting, and Participants:**

This cross-sectional, multicenter study used the history and physical (H&P) notes from unique, randomly sampled adult admissions occurring between January 1, 2016, and December 31, 2021, at 13 hospitals in the University of Maryland Medical System (UMMS) and the John Hopkins (Hopkins) health care system to compare the performance of an LLM (GPT-4-Turbo) using zero-shot learning and prompting against humans in identifying patients with recent LTCF exposure. LLM analyses were conducted from August to September 2024.

**Exposure:**

Recent (≤12 months) LTCF exposure documented in the H&P note, as adjudicated by (1) humans and (2) an LLM.

**Main Outcomes and Measures:**

LLM sensitivity and specificity with Clopper-Pearson 95% CIs. Secondary outcomes were note review time and cost. The LLM was also prompted to provide a rationale and supporting note-text for each classification.

**Results:**

The study included 359 601 eligible adult admissions, of which 2087 randomly sampled H&P notes were manually reviewed at UMMS (1020 individuals; median [IQR] age, 58 [41-71] years; 493 [48%] male) and Hopkins (1067 individuals; median [IQR] age, 58 [48-67] years; 561 [53%] male) for LTCF residence. Compared with human review, the LLM achieved a sensitivity of 97% (95% CI, 91%-100%) and a specificity of 98% (95% CI, 97%-99%) at UMMS, and 96% (95% CI, 86%-100%) and 93% (95% CI, 92%-95%) sensitivity and specificity, respectively, at Hopkins; specificity at Hopkins improved with prompt revision (96% [95% CI, 95%-97%]). Of 117 manually reviewed LLM rationales, all were factually correct and quoted note-text accurately, and some demonstrated inferential logic and external knowledge. The LLM identified 37 (1.8%) human errors. Human review time had a mean of 2.5 minutes and cost $0.63 to $0.83 per note vs a mean of 4 to 6 seconds and $0.03 per note for LLM review.

**Conclusions and Relevance:**

In this 13-hospital study of 2087 adult admissions, an LLM accurately identified LTCF residence from H&P notes and was more than 25 times faster and 20 times less expensive than human review.

## Key Points

**Question:**

Can large language models (LLMs) identify patients who lived in a long-term care facility (LTCF) in the preceding year, and are therefore at high antimicrobial resistance risk, from free-text admission histories?

**Findings:**

In this cross-sectional study using admission histories from 2087 patients treated at 13 hospitals in 2 health care systems, compared with human review an LLM demonstrated sensitivity of 96% or greater and specificity of 93% or greater for identifying LTCF residence, demonstrated cogent reasoning, and found human errors. Human review took an average of 2.5 minutes per note and cost $0.63 to $0.83 per note vs 4 to 6 seconds and $0.03 for LLM review.

**Meaning:**

In this study, an LLM accurately identified patients with recent LTCF residence and was more than 25 times faster and 20 times less expensive than human review.

## Teaser

This cross-sectional study evaluates whether a large language model (LLM) can use unstructured health record notes to accurately identify adult patients who had past-year residence in a long-term care facility.

## Introduction

Each year in the United States, nearly 3 million people develop—and more than 35 000 individuals die from—antimicrobial-resistant infections.^1^ Hospitalized patients face some of the highest risks of developing and dying from antimicrobial-resistant infections due to frequent underlying illnesses, receipt of invasive devices and surgical procedures during hospitalization, and the high prevalence of antimicrobial-resistant organisms in hospital settings.^2^

A core strategy for reducing the toll of antimicrobial resistance in US hospitals is targeted screening of high-risk patients for asymptomatic colonization.^3^ When colonized patients are identified early, infection control teams can intervene to prevent transmission,^4^ and clinicians can provide tailored care to reduce infection risk and optimize treatment of infections that do occur.^5^ Among US hospitalized patients, one of the most important colonization risk factors is residence or a recent stay in a long-term care facility (LTCF),^3,6,7^ with up to half of LTCF residents colonized with at least one multidrug-resistant bacterial organism.^8^ Yet, identifying whether an inpatient has recent LTCF exposure remains extremely challenging. Few states maintain databases for tracking patients who receive care at LTCFs,^9^ and most hospitals do not formally assess patients for LTCF exposure (eg, via intake screening). Moreover, most LTCF patients do not have an LTCF admission source coded in the electronic health record (EHR) for their admission; many do not have recent discharges to LTCFs recorded either.^10,11^ Instead, clinicians often document LTCF exposure within the history and physical (H&P) note.^10^ However, because H&P notes are free-text (ie, unstructured), absent manual medical record review, which is resource-intensive and cannot be incorporated into automated algorithms, or natural language processing (NLP) techniques for analyzing free-text data, this LTCF information has historically been unusable.

Generative artificial intelligence (AI)–based large language models (LLMs) offer a promising, emerging alternative to manual medical record review and traditional natural language processing. LLMs excel at analyzing free-text data, and they offer several practical advantages compared with traditional NLP, namely, that they require minimal data preprocessing and can provide justifications for classifications. However, LLMs raise new challenges, including stochastic outputs and the potential for factual errors.^12,13,14^ Additionally, data evaluating LLM performance on the complexity of clinical notes containing protected health information (PHI), not deidentified or curated data sources, remain extremely limited. Therefore, the objective of the current study was to evaluate the performance of an LLM for identifying high-risk, LTCF-exposed patients from identifiable clinical notes, across 2 independent health systems.

## Methods

### Study Setting and Population

We conducted a retrospective study of admissions across 2 health care systems in Maryland. The first cohort (UMMS cohort) included randomly sampled, unique adult admissions from January 1, 2016, to December 31, 2021, across 12 acute care hospitals in the University of Maryland Medical System (UMMS). UMMS hospitals are distributed across the state of Maryland. The second cohort (Hopkins cohort*)* included randomly sampled, unique adult admissions from July 1, 2016, to December 31, 2018, among patients admitted to an intensive care unit (ICU) during hospitalization at The Johns Hopkins Hospital in Baltimore, Maryland. Both sites use Epic EHRs, and for each included admission we extracted the admission H&P note and patient demographic and hospital data. The University of Maryland School of Medicine and The Johns Hopkins School of Medicine institutional review boards approved this study, with a waiver of informed consent due to the impracticality of contacting the large number of patients as well as the time interval between the study and the original admissions. This study followed the Strengthening the Reporting of Observational Studies in Epidemiology (STROBE) reporting guideline for cross-sectional studies.

### Manual Human Review of H&P Notes for LTCF Exposure

Using standardized guidelines developed through expert consensus (K.E.G., P.D.T., A.D.H., and E.Y.K.) (eMethods 1 in Supplement 1), at each study site trained reviewers manually reviewed and classified each note for mention of LTCF exposure (ie, residence) in the 12 months before admission. Consistent with National Healthcare Safety Network LTCF guidelines,^15^ exposure included permanent (eg, assisted living) and short-term (eg, postacute skilled nursing facility) residence. At UMMS, notes from 50 randomly sampled admissions were reviewed by both K.E.G. and M.T., and chance-corrected interrater agreement was calculated using the Cohen κ statistic.^16^ Based on the high agreement identified (Cohen κ = 0.85), the remaining notes were divided and reviewed individually (K.E.G. and M.T.). At Hopkins, notes were reviewed individually (P.B.-V., S.F., N.K., J.H.L., and A.H.V.). Reviewers maintained logs to record note review times and met regularly to discuss and adjudicate uncertainties. Final sample sizes were determined by resource availability for manual review.

### LLM Evaluation for Ascertaining LTCF Exposure From H&P Notes

Both the University of Maryland and the Johns Hopkins Health System deploy cloud-based, secure research enclaves for computing on PHI-containing data (Microsoft Azure). H&P notes were passed to the LLM (GPT-4 Turbo [Open AI]; temperature, 0; implementing model, “gpt-4-0125-preview” at UMMS and “gpt-4-turbo-2024-04-09” at Hopkins) via Python using the OpenAI applied programming interface within each institution’s secure computing environment. Using zero-shot learning and prompting (ie, neither the LLM nor the prompt was fine-tuned or otherwise refined on training examples), at each site we prompted the LLM with the *original prompt *as follows:

You are a physician tasked with evaluating whether the note written about a patient in the hospital suggests that the patient was recently in a long-term care facility, defined as within the year before being admitted to the hospital. Long-term care facilities include assisted living facilities, skilled nursing facilities, non-behavioral rehabilitation facilities, nursing homes, and group homes that assist with activities of daily living (ADLs). Users will provide a hospital admission note and you will respond with a yes or no as a JSON object, reflecting your best guess whether the patient has recent long-term care facility exposure. Additionally, add the reasoning for your yes or no response, and provide relevant text from the note in quotation marks that justified your decision. Here’s an example of your output format: { “long-term care facility”: “”,”reasoning”: “”} [*Full H&P note was appended to the prompt*]

LLM classification accuracy was compared with criterion-standard human review. On the UMMS cohort all LLM-human discordant classifications (ie, LLM false positives and false negatives) were reviewed for common or thematic mistakes. Based on this review, the original prompt was revised (*revised prompt*; eMethods 2 in Supplement 1); the revised prompt was reapplied only on the full Hopkins cohort; and performance was reassessed. All remaining LLM-human discordant results from this second run were manually reviewed. All analyses used new sessions to prevent residual contamination or model learning across runs.

### Subanalyses Evaluating LLM Consistency, Reasoning, and Comparison With Structured EHR Data

**Figure 1.**
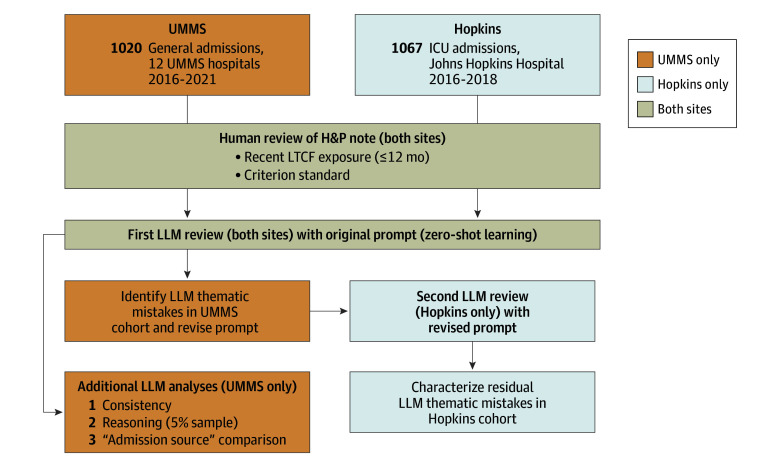
Flowchart for the Evaluation of Human vs Large Language Model (LLM) Classification of Long-Term Care Facility (LTCF) Exposure From History and Physical (H&P) Clinical Notes ICU indicates intensive care unit; UMMS, University of Maryland Medical System.

Three additional analyses on the UMMS cohort were performed. First, because LLMs are probabilistic, output may vary across otherwise-identical runs. To assess LLM consistency, the original prompt was applied once per day, for 5 consecutive days, and classification concordance across runs using Fleiss κ was calculated.^17^ Second, on a 5% subsample of the UMMS cohort selected through stratified-random sampling (a 1:1 ratio of randomly selected LTCF-positive and LTCF-negative notes based on criterion-standard review), the factual accuracy of the LLM-provided rationales was assessed by manually comparing the reasoning and quoted text with the H&P note. Third, to quantify the relative yield of LLM ascertainment, the proportion of LTCF-exposed patients who had an LTCF admission source in the EHR^11^ was calculated (Figure 1).

### Statistical Analysis

Descriptive statistics were calculated using mean (SD), median (IQR), and frequency count (percentage). LLM performance was assessed by calculating sensitivity and specificity compared with human review with exact Clopper-Pearson 95% CIs. The McNemar test for correlated proportions was used to assess the difference in LLM performance between the original and revised prompts on the Hopkins cohort. For all statistics, results were considered significant at *P* < .05 in 2-sided tests. Analyses were performed using Python versions 3.10.11 and 3.12.0 (Python Software Foundation). LLM analyses were conducted between August and September 2024.

## Results

During the study period, there were 359 601 eligible adult admissions, of which 2087 randomly sampled H&P notes were manually reviewed for recent LTCF exposure: 1020 general inpatient admissions in the UMMS cohort (median [IQR] age, 58 [41-71] years; 493 [48%] male), which drew from all 12 UMMS hospitals and 2016 to 2021 study years, and 1067 admissions in the Hopkins cohort (median [IQR] age, 58 [48-67] years; 561 [53%] male), which drew from inpatients admitted to an ICU during hospitalization at the Johns Hopkins Hospital from 2016 to 2018. At UMMS and Hopkins, 45% and 54%, respectively, of study patients identified as belonging to a minoritized racial and ethnic group. Specifically, based on EHR data at UMMS, 381 patients (37%) were Black; 57 (6%) belonged to additional racial groups (ie, American Indian or Alaskan Native, Native Hawaiian or Other Pacific Islander, unknown, or another race); and 24 (2%) were Asian. At Hopkins, 457 (43%) were Black; 92 (9%) belonged to additional racial groups, and 27 (3%) were Asian (Table 1). By human (criterion-standard) review, the prevalence of LTCF exposure across all H&P notes was 7% (76 patients) in the UMMS cohort and 5% (48 patients) in the Hopkins cohort.

**Table 1.** Cohort Characteristics for 1020 Randomly Sampled Admissions Across 12 Hospitals in UMMS (2016-2021) and 1067 Randomly Sampled Admissions From Patients Admitted to an Intensive Care Unit at Hopkins (2016-2018)

Characteristic	Cohort, No. (%)	
	UMMS (n = 1020)	Hopkins (n = 1067)
Age, median (IQR), y	58 (41-71)	58 (45-67)
Sex		
Female	527 (52)	506 (47)
Male	493 (48)	561 (53)
Race^a^		
Asian	24 (2)	27 (3)
Black	381 (37)	457 (43)
White	558 (55)	491 (46)
Additional group^b^	57 (6)	92 (9)
Admission year^c^		
2016	146 (14)	463 (43)
2017	163 (16)	536 (50)
2018	145 (14)	68 (6)
2019	184 (18)	0
2020	183 (18)	0
2021	199 (20)	0
Hospital location		
Urban	509 (50)	1067 (100)
Suburban	430 (42)	0
Rural	81 (8)	0

Abbreviation: UMMS, University of Maryland Medical System.

^a^ Derived from the patient race field in the UMMS and Johns Hopkins Health System electronic health record systems.

^b^ Includes American Indian or Alaskan Native, Native Hawaiian or Other Pacific Islander, Unknown, and other race, which was not further defined.

^c^ Restricting to the first admission per patient during the study period.

### LLM Performance: Original Prompt

Using the original prompt (reflecting a zero-shot learning and prompting approach), the LLM achieved a sensitivity and specificity on the UMMS general inpatient cohort of 97% (95% CI, 91%-100%) and 98% (95% CI, 97%-99%), respectively, across 1017 notes; 3 of 1020 H&P notes (0.3%) could not be processed because their content triggered LLM safety filters for violence or self-harm. At 7.5% LTCF prevalence, this correlated with positive and negative predictive values of 78% (95% CI, 70%-84%) and 100% (95% CI, 99%-100%), respectively. On the Hopkins cohort, LLM sensitivity was 96% (95% CI, 86%-100%), and specificity was 93% (95% CI, 92%-95%) (Table 2).

**Table 2.** Performance Characteristics of an LLM for Classifying History and Physical Clinical Notes for Mention of Recent (≤12 Months) LTCF Exposure

Outcome	Point estimate (95% CI), %		
	Original prompt		Hopkins (revised prompt) (n = 1067)
	UMMS (n = 1017)^a^	Hopkins (n = 1067)	
LTCF prevalence by human review, No./total No. (%)	76/1017 (7.5)	48/1067 (4.5)	48/1067 (4.5)
Overall accuracy	98 (97-99)^b^	93 (86-99)^c^	96 (95-97)^c^
Sensitivity	97 (91-100)	96 (86-100)	94 (83-99)
Specificity	98 (97-99)	93 (92-95)	96 (95-97)
PPV	78 (70-84)^b^	40 (35-46)^c^	52 (45-60)^c^
NPV	100 (99-100)^b^	100 (99-100)^c^	100 (99-100)^c^
Length of LLM-provided rationales, median (IQR)^d^			
Characters	396 (263-408)	390 (346-439)	428 (376-466)
Tokens	86 (70-103)	89 (79-100)	96 (87-104)
Computing cost, mean, $/note	0.03	0.03	0.03
Computing time, mean, s/note	5.8^e^	4.2	4.4
Classification performance consistency^f^	0.98	NA	NA

Abbreviations: LLM, large language model; LTCF, long-term care facility; NA, not applicable; NPV, negative predictive value; PPV, positive predictive value; UMMS, University of Maryland Medical System.

^a^ Overall, 3 of 1020 notes were excluded due to triggering the LLM safety content filter for mention of violence or self-harm.

^b^ At an LTCF prevalence of 7.5%.

^c^ At an LTCF prevalence of 4.5%.

^d^ Excluding spaces. Tokens were estimated using the Python ‘TikToken’ package.

^e^ Processing time was longer per note at UMMS because each output was iteratively written to a CSV file.

^f^ Estimated by the Fleiss κ for interrater agreement based on concordance across 5 independent runs, executed once per day for 5 consecutive days. In each run, the same 3 notes triggered the safety content filter. The Fleiss κ value of 0.98 indicates near-perfect agreement in LLM classifications across runs (ie, extremely low variability).

Re-executing the original prompt an additional 5 times on the UMMS cohort, LLM classification accuracy ranged from 97.5% to 97.9%. The Fleiss κ value for interrater agreement was 0.98, indicating near-perfect classification agreement across runs (Table 2).^18^ Compared with the LLM’s detection of 97% of LTCF-positive admissions, the EHR admission source field would have detected 33% of patients (25) with LTCF exposure in the UMMS cohort, plus an additional 5 patients with LTCF admission sources that were negative by human review of the H&P note.

### Thematic Review of LLM Errors (UMMS Cohort), Prompt Revision, and LLM Performance Using Revised Prompt (Hopkins Cohort)

**Figure 2.**
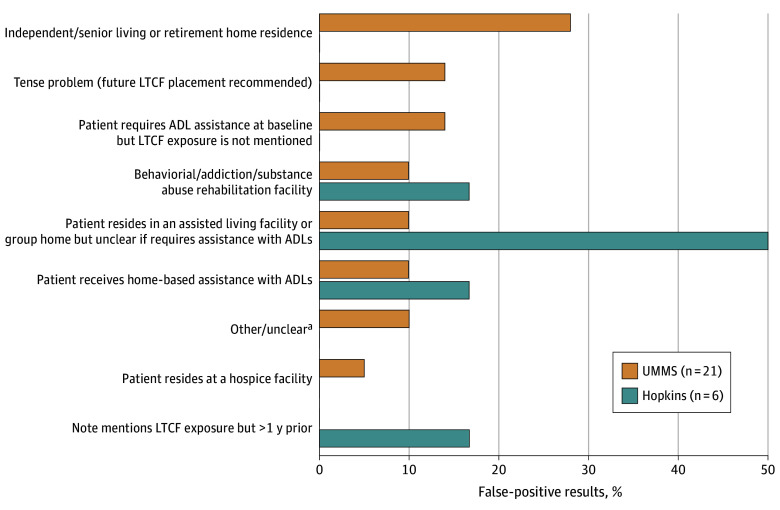
Distribution of Large Language Model False-Positives at University of Maryland Medical System (UMMS) Using the Original Prompt and at Hopkins Using the Revised Prompt, by Type of Error This figure includes ambiguous classifications (eFigure 1 in Supplement 1) but does not include the 35 large language model classifications that were initially marked as false positives at Hopkins that were determined to be human errors. ADL indicates activity of daily living; LTCF, long-term care facility. ^a^Other/unclear includes 1 instance where the note was internally inconsistent and 1 instance where the note indicated that in a recent, prior admission the treating clinician had considered discharge to a rehabilitation facility but follow-up was unclear.

In the UMMS cohort, the LLM produced 2 false-negative and 21 false-positive results compared with human review. Of the 2 false negatives, 1 reflected a missed LTCF acronym in the context of clinical uncertainty, and the other resulted from the LLM’s failure to identify an abbreviated/informal proper name of an LTCF facility. Of the 21 false positives, the most common thematic errors involved residence in senior independent living and retirement facilities (6 errors [29%]), recommendation for future rehabilitation or assisted living placement (3 errors [14%]), a baseline functional status requiring assistance with ADLs but not explicitly mentioning LTCF exposure (3 errors [14%]), and stays in substance abuse facilities and group recovery homes (2 errors [10%]) (Figure 2).

Based on these errors in the UMMS cohort, we revised the original prompt to clarify exposures and descriptors that do or do not qualify as LTCF exposure (eMethods 2 in Supplement 1) and executed the revised prompt on the full Hopkins cohort (which served as a held-out test set). The revised prompt achieved a sensitivity of 94% (95% CI, 83%-99%) (2–percentage point decrease from original prompt), a specificity of 96% (95% CI, 95%-97%) (3–percentage point increase from original prompt), and an overall accuracy rate of 96% (95% CI, 95%-97%) (3–percentage point increase from original prompt; *P* < .001 for accuracy difference) (Table 2). At 4.5% LTCF prevalence, the positive and negative predictive values were 52% (95% CI, 45%-60%) and 100% (95% CI, 99%-100%), respectively (eTable in Supplement 1). Figure 2 outlines the errors resulting from this second execution. On rereview, 35 of 41 false positives (85%) and 2 of 3 false negatives (67%) were determined to be human errors, ie, the LLM’s classification was correct (eFigure in Supplement 1). Because we only rereviewed human-LLM discordant observations and not the full note set, however, we did not adjust LLM sensitivity and specificity estimates for these human errors.

### LLM-Provided Rationales

For each note, the LLM provided an explanation for its yes/no LTCF classification, which was generally a few sentences long (Table 2). Table 3 provides an illustrative sample of LLM-provided rationales, including the LLM’s knowledge of LTCF acronyms not included in the prompts; identification of internal note inconsistencies; and successful interpretation of flowsheet copy-and-pasted content. The LLM also appeared to perform inferential logic. For example, sometimes the LLM justified an LTCF-unexposed classification by referencing patient characteristics incompatible with LTCF residence—eg, “the patient was found alone at a restaurant, which implies he was living independently or at least not in a care facility at the time of the incident. There is no mention of a recent stay in any type of long-term care facility in the history provided.” In another instance, the LLM classified recent LTCF exposure, which although incorrect was logical: “[T]he patient was ‘found by caregiver this am with some confusion’… The presence of a caregiver, especially in the context of managing the patient’s medications and responding to her acute condition, supports the possibility of recent long-term care facility exposure.” In manual review of a 5% subsample of the UMMS cohort (50 H&P notes), plus the human-discordant LLM results manually reviewed at each site (67 H&P notes), no factual errors were identified, and the LLM quoted all note text accurately. However, for one note, the LLM assumed a residential location for an accidental injury that was implied but not explicitly stated (eResults in Supplement 1).

**Table 3.** Examples of LLM-Provided Rationales and Supporting Note Text

Demonstrating	LLM rationale	Relevant text fragment from H&P note
Knowledge of LTCF acronyms (“TCC”) not included in prompts	“The note mentions that the patient is ‘presenting to us from TCC for non-compliance and hyperglycemia.’ TCC likely refers to a Transitional Care Center, which is a type of facility that provides care to individuals transitioning from hospital to home or a long-term care facility. This suggests recent exposure to a long-term care setting.”	“…The patient is now status post DDT on [redacted] from [redacted]. He received Campath for induction and experienced immediate graft function. There is not a history of rejection. There has never been an allograft biopsy. The patient’s clinical course has been: complicated by worsening DM. He is now presenting to us from TCC for non-compliance and hyperglycemia. No fever, nausea, diarrhea, SOB, chest pain or urinary symptoms…”
Inferential logic	“The note does not provide any direct information or hints that suggest the patient was recently in a long-term care facility. The patient was found alone at a restaurant, which implies [redacted] was living independently or at least not in a care facility at the time of the incident. There is no mention of a recent stay in any type of long-term care facility in the history provided.”	“…[Redacted] is a [redacted] y.o. [redacted] who presents with syncope. The patient was eating alone at a restaurant when one of the workers noticed that the patient appeared to be asleep. When the patient could not be easily aroused EMS was called. The patient began to respond in the ambulance but [redacted] arousal was punctuated by vomiting…”
Successful interpretation of drop-down questionnaire content (“X” response) copy and pasted by EHR into H&P note	“The note explicitly states under ‘SOCIAL HISTORY’ that the patient resides ‘Independently’. There is no mention of recent or current residence in a long-term care facility within the year before being admitted to the hospital.”	“…Ã’Â¶ Ã’Â¶SOCIAL HISTORY: Ã’Â¶Patient resides: Ã’Â¶Independently X Ã’Â¶Assisted Living Ã’Â¶SNF…”
Identification of possible internal note inconsistency^a^	“The note mentions ‘Information from rehab medication report’ and ‘Information from rehab medication sheet’ under the Allergies section, which suggests that the patient has been in a rehabilitation facility, which falls under the category of a long-term care facility, within the timeframe considered.”	“… ¶ Ã’Â¶Patient has a caretaker that lives with her at home, notes of medication non-compliance in the past… … ¶Allergies Ã’Â¶Allergen Reactions Ã’Â¶Ã¢â’¬Â¢ [redacted] Cough Ã’Â¶Ã¢â’¬Â¢ Anesthetics, [redacted] Ã’Â¶ [redacted] Ã’Â¶Ã¢â’¬Â¢ [redacted] Ã’Â¶ Information from rehab medication report Ã’Â¶Ã¢â’¬Â¢ [redacted] Ã’Â¶ Information from rehab medication sheet Ã’Â¶”

Abbreviations: DDT, deceased donor transplantation; DM, diabetes mellitus; EHR, electronic health record; EMS, emergency medical services; H&P, history and physical; LLM, large language model; LTCF, long-term care facility; SNF, skilled nursing facility; SOB, shortness of breath; y.o., years old.

^a^ Human review classified the note as no recent LTCF exposure based on the early statement that the patient lives at home. The LLM identified that the allergy information had come from a rehabilitation facility medication sheet, suggesting that the patient may have recently been LTCF-exposed. However, the LLM’s rationale did not note the statement about the patient living at home.

### Resource Utilization for Human vs LLM Review

Human review of the H&P notes took a mean of 2.5 minutes per note at both sites. At $15 to $20 hourly pay for research assistants, the cost of human review averaged $0.63 to $0.83 per note, excluding employer taxes, onboarding, and training time. LLM computing is charged by the number of input (prompt + H&P note) and output (LTCF classification + rationale) tokens, and had a mean cost of $0.03 per note at both UMMS and Hopkins. LLM computing time had a mean time of 5.8 seconds per note at UMMS and 4.2 to 4.4 seconds per note at Hopkins (Table 2). Taken together, the LLM was more than 25 times faster and 20 times less expensive than human review for classifying LTCF exposure from the H&P note.

## Discussion

To our knowledge, this is the first study to evaluate LLMs for ascertaining LTCF exposure from clinical notes and one of the only multisite LLM evaluations on PHI-containing patient data. Across a diverse, multicenter cohort of patients admitted to 13 hospitals between 2016 and 2021, an off-the-shelf LLM accurately identified LTCF exposure from H&P notes even without dedicated pretraining for this task. Moreover, the LLM’s rationales for LTCF classifications were accurate and cogent, and the LLM was substantially faster and cheaper than human review. Taken together, these findings suggest that LLMs could substantially improve the identification of LTCF-exposed inpatients to combat the spread of antimicrobial resistance in US hospitals.

We designed our evaluation to provide evidence relevant to practicing clinicians and hospital epidemiologists. Therefore, because most institutions have not manually reviewed notes for LTCF exposure, we first evaluated the LLM using a zero-shot learning and prompting approach, in which neither the LLM nor the prompt was fine-tuned or otherwise refined on training examples. In this first-pass assessment, the LLM achieved a sensitivity and specificity of 97% and 98%, respectively, at UMMS and 96% and 93%, respectively, at Hopkins. Reviewing the LLM errors at UMMS and revising the prompt further improved accuracy on the Hopkins cohort (applying the revised prompt to UMMS data would have artificially improved performance, akin to overfitting for traditional prediction models). Nevertheless, sensitivity and specificity remained lower on the Hopkins cohort, even after revision. It is possible that site differences in population and disparate model versions contributed to this performance differential. However, the most likely reason was an imperfect criterion standard, because we found that 35 of 41 LLM responses that were marked as false positives at Hopkins were actually correct classifications. Had we adjusted LLM performance estimates for these human errors, LLM specificity would have exceeded the 96% reported.

Beyond a yes-or-no LTCF classification, we prompted the LLM to provide the reasoning for the decision and text from the note to support the rationale. Across 117 rationales that were manually reviewed, all were factually correct, and all note text was quoted accurately. More surprising, however, was that these rationales included statements consistent with inferential logic. The LLM used contextual details (location of injury, presence of caregiver at awakening) to make logical conclusions about whether the patient had recent LTCF exposure.

Whether the LLM was reasoning when producing its rationales is a question of academic controversy,^19,20^ and more research across a larger set of rationales is warranted. Yet notwithstanding these caveats, we envision rationales becoming an important component of LLM deployments. Even highly accurate LLMs will make errors, and rationales enable users to understand why the LLM made mistakes and what its operating assumptions were. Moreover, even when programs maintain some manual review, LLM-provided classifications and rationales could streamline processes and reduce errors. When LLMs demonstrate high sensitivity and negative predictive values, as occurred in this study, they could perform first-pass reviews to winnow the records requiring human assessment. Similarly, even for fully manual reviews, LLMs could offer a parallel review to catch human mistakes, with human adjudication of discrepancies. In both instances, we expect appropriately validated rationales would accelerate these reviews or adjudications, because we found it substantially faster to read LLM rationales—effectively, high-level note summaries—compared with full notes.

### Strengths and Limitations

As a multicenter study spanning multiple years, health care systems, hospitals, and general inpatient and ICU cohorts, our study benefitted from a large, diverse sample to evaluate LLM performance and increase study generalizability. Our use of PHI-containing notes, rare in the LLM literature, was also essential, because notes may reference LTCFs by proper facility names (which are removed during deidentification).

However, our study is also subject to several limitations. First, we only evaluated 1 LLM that our institutions have approved for secure deployment and did not use methods that could further improve performance, such as fine-tuning and retrieval augmented generation.^21^ Evaluating a broader suite of LLMs and prompting techniques would be an important area of future research, as would evaluation in different geographic regions. Second, we could not evaluate the same LLM version at each site because of technical limitations, although both were versions of the same model and thus unlikely to be a substantial contributor to site performance differences. Third, because of cost and resource limitations, each note at Hopkins was only reviewed by a single research assistant, which likely led to a higher rate of human error but demonstrates the practical potential of LLMs for identifying human mistakes. Fourth, this study evaluated whether LLMs could identify LTCF exposure from H&P notes, but because H&P notes have high specificity but imperfect sensitivity for LTCF exposure,^10,11^ both human and LLM review will have missed LTCF-exposed patients whose notes did not mention LTCF exposure. Future work is planned to evaluate additional EHR data sources and to quantify how much LLM-extracted risk factors improve detection of colonized patients.

## Conclusions

Across 13 hospitals in 2 independent health care systems, we found that LLMs were accurate, fast, and inexpensive for identifying LTCF-exposed patients from admission histories. Although further validation across additional institutions is needed, we view LLMs as a highly promising strategy for extracting this important antimicrobial resistance risk factor from free-text EHR data. At both UMMS and Hopkins, this analysis represented the first large-scale analysis of LLMs on identifiable patient notes. As more institutions implement LLMs within Health Insurance Portability and Accountability Act–compliant, secure computing environments, the feasibility of this approach across US hospitals will increase.

## Notes

Supplement 1. **eMethods 1.** Definition of Recent (≤ 12 Months Before Admission) Long-term Care Facility (LTCF) Exposure **eMethods 2.** Revised Prompt Applied Only to the Hopkins Cohort **eResults.** Additional Information Regarding the LLM’s Assumption of a Residential Injury Location (From Manual Review of 117 LLM-Provided Rationales) **eTable.** Characteristics of LLM Errors on the Hopkins Cohort That Were Determined to be Human Errors (i.e., the LLM Was Correct) **eFigure.** Characteristics of LLM Errors at UMMS (Original Prompt) and at Hopkins after Prompt Revision (Revised Prompt)

Supplement 2.
Data Sharing Statement
