# Supplementary material for: Identification of Long-Term Care Facility Residence From Admission Notes Using Large Language Models
Source: JAMA Netw Open. 2025 May 22;8(5):e2512032. doi: 10.1001/jamanetworkopen.2025.12032 (PMC12100451; doi:10.1001/jamanetworkopen.2025.12032)
Supplement: Supplement 1. — eMethods 1. Definition of Recent (≤ 12 Months Before Admission) Long-term Care Facility (LTCF) Exposure eMethods 2. Revised Prompt Applied Only to the Hopkins Cohort eResults. Additional Information Regarding the LLM’s Assumption of a Residential Injury Location (From Manual Review of 117 LLM-Provided Rationales) eTable. Characteristics of LLM Errors on the Hopkins Cohort That Were Determined to be Human Errors (i.e., the LLM Was Correct) eFigure. Characteristics of LLM Errors at UMMS (Original Prompt) and at Hopkins after Prompt Revision (Revised Prompt) [file jamanetwopen-e2512032-s001.pdf]

Supplemental Online Content

Goodman KE, Robinson ML, Shams SM, et al. Identification of long-term care facility residence from admission notes using large language models. *JAMA Netw Open.* 2025;8(5):e2512032. doi:10.1001/jamanetworkopen.2025.12032

eMethods 1. Definition of Recent (≤ 12 Months Before Admission) Long-term Care Facility (LTCF) Exposure

eMethods 2. Revised Prompt Applied Only to the Hopkins Cohort

eResults. Additional Information Regarding the LLM's Assumption of a Residential Injury Location (From Manual Review of 117 LLM-Provided Rationales)

eTable. Characteristics of LLM Errors on the Hopkins Cohort That Were Determined to be Human Errors (i.e., the LLM Was Correct)

eFigure. Characteristics of LLM Errors at UMMS (Original Prompt) and at Hopkins after Prompt Revision (Revised Prompt)

This supplemental material has been provided by the authors to give readers additional information about their work.

© 2025 Goodman KE et al. *JAMA Network Open*.

eMethods 1. Definition of Recent (≤ 12 Months Before Admission) Long-term Care Facility (LTCF) Exposure

Human reviewers were instructed to classify a history & physical (H&P) note as positive for recent LTCF exposure if any of the following criteria were met. When exposure timing was unclear, reviewers were instructed to use their best judgment.

Mention of permanent or short-term (i.e., a recent stay) residence in a:

- Long-term care facility
- Nursing home
- Skilled nursing facility (SNF)
- Assisted living facility (ALF)
- Rehabilitation facility (non-behavioral, non-addiction-related)
- Chronic facility
- "Other subacute" facility
- Facilities for patients with severe cognitive deficits who need assistance with activities of daily living (ADLs)

© 2025 Goodman KE et al. *JAMA Network Open*.

eMethods 2. Revised Prompt Applied Only to the Hopkins Cohort

You are a physician tasked with evaluating whether the note written about a patient in the hospital suggests that the patient was recently in a long-term care facility, defined as within the year before being admitted to the hospital. Long-term care facilities include assisted living facilities (ALFs), skilled nursing facilities, rehabilitation facilities (not for substance, alcohol, or drug abuse), nursing homes, and group homes that assist with activities of daily living (ADLs). Senior living, independent living, and retirement communities do not qualify as LTCFs unless the note indicates the patient receives assistance with daily living activities. Similarly, if the patient receives daily living assistance outside of an LTCF (e.g., at home), that does not qualify as LTCF exposure, and neither does hospice care. Finally, if the patient is recommended for future LTCF care but it did not occur prior to the current admission, that does not qualify as LTCF exposure because it has not happened yet. Users will provide a hospital admission note and you will respond with a yes or no as a JSON object, reflecting your best guess whether the patient has had recent long-term care facility exposure. Additionally, add the reasoning for your yes or no response, and provide relevant text from the note in quotation marks that justified your decision. Here's an example of your output format:
{ "long-term care facility": "","reasoning": ""}

© 2025 Goodman KE et al. *JAMA Network Open*.

eResults. Additional Information Regarding the LLM's Assumption of a Residential Injury Location (From Manual Review of 117 LLM-Provided Rationales)

The H&P note stated that the patient: "Was walking down his stairs, which are under construction, when he slipped."

The LLM explained that: "The history of present illness indicates the injury occurred *at home*, specifically mentioning the patient was walking down stairs under construction *at his residence*" (emphasis added).

The note did not explicitly specify that the patient's fall occurred at home or at his residence, despite specifying that the accident occurred on "his" stairs.

© 2025 Goodman KE et al. *JAMA Network Open*.

eTable. Characteristics of LLM Errors on the Hopkins Cohort That Were Determined to be Human Errors (i.e., the LLM Was Correct)

Human Errors	Count
**LLM "False-negative" (i.e., human incorrectly identified the patient as LTCF-exposed)**	
Human reviewer incorrectly interpreted pulmonary rehab to be a LTCF	2
**LLM "False-positive" (i.e., human incorrectly stated the patient was *not* LTCF-exposed)**	
Human reviewer missed mention of prior discharge to LTCF within 12 months of presentation	27
Human reviewer missed current living status at LTCF	5
Patient lives in a group home where the reader may appropriately infer that assistance with ADLs is provided	3

© 2025 Goodman KE et al. *JAMA Network Open*.

eFigure. Characteristics of LLM Errors at UMMS (Original Prompt) and at Hopkins after Prompt Revision (Revised Prompt)

eFigure 1 reflects LLM false-positive and false-negative results, which were manually reviewed by K.G. and M.R. Ambiguous classifications reflect instances where K.G. and M.R. agreed with the human classification on balance but acknowledged that an LTCF-positive or LTCF-negative classification would have been defensible. Human errors in the third column reflect instances where, upon re-review, the LLM was determined to be clearly correct. Please see Supplemental Table 1, above, for a more detailed description of these human error types.

	Total No.	Were any classifications ambiguous? n (row %)	Were any classifications clear human errors on re-review? n (row %)
**UMMS Original Prompt** n=1017[a]			
False-Positives	21	Yes – 5 (24%)	No
False-Negatives	2	No	No
**Hopkins Revised Prompt** n=1067			
False-Positives	41	Yes – 1 (2%)	Yes – 35 (85%)
False-Negatives	3	Yes – 1 (33%)	Yes – 2 (66%)

Abbreviations: UMMS, University of Maryland Medical System.

[a] 3 of 1020 notes could not be processed due to their content triggering LLM safety content filters for mention of violence or self-harm.

© 2025 Goodman KE et al. *JAMA Network Open*.
