# Supplementary material for: Identification of Long-Term Care Facility Residence From Admission Notes Using Large Language Models
Source: JAMA Netw Open. 2025 May 22;8(5):e2512032. doi: 10.1001/jamanetworkopen.2025.12032 (PMC12100451; doi:10.1001/jamanetworkopen.2025.12032)
Supplement: Supplement 2. — Data Sharing Statement [file jamanetwopen-e2512032-s002.pdf]

Data Sharing Statement

Goodman. Identification of Long-Term Care Facility Residence From Admission Notes Using Large Language Models. *JAMA Netw Open*. Published May 22, 2025. doi:10.1001/jamanetworkopen.2025.12032

Data
Data available: No

Additional Information
Explanation for why data not available: The study uses identifiable patient notes, which are prohibited from data sharing due to HIPAA.
